# [^18^F]FDG PET/CT for treatment monitoring and prediction of progression in retroperitoneal fibrosis

**DOI:** 10.1007/s00259-025-07479-6

**Published:** 2025-08-14

**Authors:** Christian Bayerl, Jonas Kaufmann, Giulia Metzger, Julian M. M. Rogasch, Holger Amthauer, Imke Schatka, Winfried Brenner, Markus van der Giet, Christian Furth

**Affiliations:** 1 Department of Nuclear Medicine, Charité – Universitätsmedizin Berlin, corporate member of Freie Universität Berlin and Humboldt-Universität zu Berlin, Berlin, Germany; 2 National Center for Tumor Diseases (NCT), Campus Berlin, Berlin, Germany; 3 German Cancer Consortium (DKTK), Campus Berlin, Berlin, Germany; 4 Department of Nephrology, Charité – Universitätsmedizin Berlin, corporate member of Freie Universität Berlin and Humboldt-Universität zu Berlin, Berlin, Germany

**Keywords:** Retroperitoneal fibrosis, Prednisolone, Rituximab, Metabolic active volume

## Abstract

**Purpose:**

Retroperitoneal fibrosis (RPF) is a rare inflammatory disease, that, if left untreated, can lead to ureteral obstruction and subsequent renal impairment. First-line treatment is prednisolone, with rituximab, often used for refractory cases. This study evaluates treatment response in both [^18^F]FDG PET and CT, and potential baseline parameters for early prediction of progression.

**Methods:**

50 patients with RPF underwent at least two [^18^F]FDG PET/CT scans (baseline, BL, and first follow-up, FU1), 36 patients a second (FU2), 18 patients a third follow-up (FU3). PET parameters SUV_max_, SUV_mean_, SUV_peak_, metabolic active volume (MAV), thickness (CT_rim_) and cranio-caudal extension (CT_cc_) of the retroperitoneal mass were measured. Therapy groups were divided into prednisolone, rituximab and the combination of both.

**Results:**

All PET parameters showed significant correlations with CT_rim_ at all four timepoints. After therapy all PET parameters and CT_rim_ decreased significantly (*p* ≤ 0.021). Highly significant metabolic and morphologic response was seen in the prednisolone (*p* ≤ 0.003) and the combination therapy group (*p* ≤ 0.001). At FU2, eight patients showed progression, with MAV as a good predictor of progression in BL (*p* = 0.041; 217.33 versus 100.86 ml). At FU2, SUV_max_, SUV_peak_ and MAV differed significantly between progression and non-progression group (*p* ≤ 0.009), while CT showed no significant differences.

**Conclusion:**

Our findings underscore the superiority of PET against CT in therapy monitoring of RPF, especially in the detection of progression at FU2. Higher BL MAV correlated with progression at FU2, indicating its potential as a predictive marker. Still, especially when PET is not available, CT can be considered for initial therapy monitoring.

## Introduction

Retroperitoneal fibrosis (RPF) is a rare, chronic inflammatory disease characterized by the development of fibrous tissue in the retroperitoneal space [1–3]. The precise etiology of RPF remains unclear, though it is believed to involve an autoimmune component and there is a significant overlap with IgG4-related-disease [1, 4]. Timely and accurate diagnosis, as well as monitoring of therapeutic response, are critical for effective management of RPF to prevent severe complications, such as renal failure.

Computed tomography (CT) has been the mainstay imaging modality for the diagnosis and follow-up of RPF due to its ability to delineate the extent of fibrotic tissue and detect complications [1]. However, positron emission tomography combined with computed tomography (PET/CT) using ^18^F-labelled fluorodeoxyglucose ([^18^F]FDG) has become a well-established modality by providing metabolic information for extent of disease and therapeutic response assessment in addition to anatomical details [5–9].

Despite its diagnostic value, PET/CT remains limited in availability due to economic and infrastructural constraints. This highlights the importance of exploring whether conventional CT can serve as a surrogate or complementary tool in evaluating disease activity and treatment response in RPF. Jansen et al. found a significant correlation between mass thickness in CT and visual PET assessment before therapy, while after therapy, this correlation was no longer present [7]. Regarding CE-CT (contrast-enhanced CT), it enables more accurate assessment of inflammation when performed alongside PET/CT [10], but its sensitivity for detecting active inflammation alone - particularly when assessing treatment response based on radiodensity - appears to be limited. Moroni et al. reported a good correlation between FDG uptake on PET and radiodensity in CE-CT, but also described two cases where the retroperitoneal mass showed little or no contrast enhancement, despite only partial remission and persistently elevated inflammatory markers [5]. These findings raise the question of whether CT alone is sufficient for response assessment during follow-up.

When evaluating treatment response, it is important to consider that changes in metabolic activity may vary depending on the therapeutic agent used. Standard first-line treatment of RPF typically involves prednisolone [11–13], but with a relatively high recurrence rate [12]. This often necessitates prolonged or high-dose administration of glucocorticoids, leading to increased risks of side effects, such as diabetes or hypertension. To address this issue, immunosuppressants, such as tamoxifen, cyclophosphamide or azathioprine are employed as alternative or adjunct therapies [11, 13–15]. In recent years, rituximab, a monoclonal antibody targeting CD20-positive B cells, has emerged as a promising therapeutic option for RPF. It has shown promising results in achieving disease remission and reducing dependency on long-term glucocorticoid therapy, including symptom resolution and radiographic improvement [16–19], even without concomitant glucocorticoid therapy [20].

In evaluating disease activity of retroperitoneal fibrosis, former studies mostly utilized the maximum standardized uptake value (SUV_max_) [5, 6, 8–10, 17, 19, 21], while only few used SUV_mean_ [17], metabolic volume [5] or qualitative scores with liver uptake serving as a reference [7, 21]. These parameters serve as key indicators of metabolic activity for both initial assessment and follow-up evaluation of therapy.

However, despite its common use, there is a lack of research examining the therapy responses of prednisolone and rituximab across a broader range of PET parameters through baseline and follow-up examinations, which might reveal alternative metrics that are better suited for monitoring and evaluation of treatment response.

After initially successful therapy, follow-up imaging is important to detect progression or recurrence at an early stage, as e.g. acute phase reactants alone are often not reliable [6]. Few studies addressed the prediction of clinical outcome in baseline and follow up examinations [10, 22]. For instance, Morin et al. examined baseline scans at diagnosis with two follow-ups (3.1 and 10.4 months after diagnosis) and found that persistent FDG uptake (represented by SUV_max_) present at second follow-up was associated with relapses [22]. Therefore, a prognostic parameter would be valuable for tailoring follow-up intervals or even anticipating metabolic progression, ultimately enabling the selection of the most effective mono- or combination therapy from the start to minimize the risk of recurrence.

This study aimed to (1) analyze and correlate a wide range of PET parameters and CT parameters to assess treatment response through baseline and follow-up imaging and (2) to identify a reliable baseline marker capable of predicting disease progression. This could optimize initial therapy selection, reduce progression rates, and improve long-term disease management.

## Materials and methods

### Study population

Between January 2018 and April 2024, a total of 86 patients with RPF received at least two [^18^F]FDG PET/CT scans in the department of nuclear medicine of Charité–Universitätsmedizin Berlin.

Inclusion criterion of patients with RPF was a minimum age of 18 years. Exclusion criteria were cases, where no therapy after first in-house [^18^F]FDG PET/CT scan was administered (*n* = 19), the absence of morphologically detectable and metabolically active lesions in [^18^F]FDG PET/CT (*n* = 9), therapy performed with other than prednisolone or rituximab (*n* = 4), unusual location of fibrosis with ambiguous histology (*n* = 1), concomitant vasculitis (*n* = 1), lack of compliance (*n* = 1) and follow-up PET scan with different tracers (*n* = 1). The diagnosis of retroperitoneal fibrosis (RPF) was established based on clinical presentation, laboratory tests, and imaging findings (CT, magnetic resonance imaging and [^18^F]FDG PET/CT), while histological confirmation was not routinely performed for ethical reasons.

This study is in agreement with the Declaration of Helsinki and the ethical standards of the research ethics committee of the Charité—Universitätsmedizin Berlin (EA4/035/25).

### [^18^F]FDG PET/CT imaging

Patients were advised to fast for at least six hours before examination. Scans were conducted with acquisition times of 2 to 3 min per bed position and had to include at least from base of the skull to the proximal femurs.

Patients received a scan either on a digital PET device (General Electric^®^ Healthcare, Discovery MI, Chicago, Illinois, United States; silicon photomultipliers [SiPM], 3-ring detector setting, Time of flight [TOF] capability, system sensitivity of 7.3 cps/kBq; *n* = 152) or on a non-digital PET device with photomultiplier tubes (Philips^®^, Gemini TF16 ASTONISH, Eindhoven, The Netherlands; lutetium-yttrium oxyorthosilicate scintillation crystals, TOF technology, system sensitivity of 6.6 cps/kBq, *n* = 2). Mean activity of [^18^F]FDG administered was 276.5 (range 182.8–382.4) MBq, 3.4 (range 2.4–5.8) MBq/kg at baseline (BL), 281.6 (range 180.9–373.3) MBq, 3.3 (range 2.1–5.2) MBq/kg at first follow-up (FU1), 290.7 (range 165.0–361.0) MBq, 3.4 (range 2.6–5.0) MBq/kg at second follow-up (FU2) and 289.9 (range 218.7–360.0) MBq, 3.5 (range 2.6–4.9) MBq/kg at third follow-up (FU3). Mean uptake time of 67.2 (range 56–90) minutes at BL, 68.2 (range 50–102) minutes at FU1, 64.9 (range 56–92) minutes at FU2 and 66.0 (range 55–86) minutes at FU3. Mean blood sugar level was 102.5 (range 76–203) mg/dl at BL, 103.7 (range 62–185) mg/dl at FU1, 108.7 (range 71–256) mg/dl at FU2 and 114.3 (range 82–242) mg/dl at FU3. PET raw data were reconstructed iteratively with Bayesian penalized likelihood reconstruction (GE “Q.Clear”) or using iterative reconstruction (ordered subset expectation maximization; OSEM) with TOF analysis (BLOB-OS-TF; iterations, 3; subsets, 33; filter, ‘smooth’ [kernel width, 14.1 cm; relaxation parameter, 0.7]; matrix, 144 × 144; voxel size, 4.0 × 4.0 × 4.0 mm^3^).

With both PET scanners, non-enhanced CT data were used for attenuation correction. Scatter correction, randoms correction and dead time correction were performed.

At BL 22 patients, at FU1 28 patients, at FU2 19 patients and at FU3 12 patients received contrast agent intravenously (Imeron 300, 80–120 ml) during the computed tomography (CT) scan with a delay of 90 s. The remaining patients did not receive contrast agent due to contraindications such as renal failure or previous allergic reactions.

### Image assessment

PET imaging data was assessed by one experienced, board-certified radiologist and nuclear physician resident (Reader 1) and one nuclear physician resident (Reader 2), each with > 2 years of experience in PET imaging using dedicated software (ROVER version 2.1.20, ABX, Radeberg, Germany). As a reference, a spherical region of interest (ROI) was placed in the liver parenchyma for SUV_max_liver_, SUV_mean_liver_ and SUV_peak_liver_.

For measurements of the retroperitoneal mass, a spherical or cylindric ROI was drawn fitting the mass as best as possible. The aortic lumen was included in measurements, while extravascular structures (e.g. ureter) were subtracted with the lower absolute threshold defined as SUV_mean_liver_. SUV_max_, SUV_mean_, SUV_peak_ and metabolic active volume (MAV) of the mass were obtained. Furthermore, SUV_mean_ was corrected for partial volume, which is referred to as SUV_mean_c_. For morphologic measurements, the maximum thickness of the mass in axial slices (CT_rim_) and the maximum craniocaudal extent in sagittal and coronal slices (CT_cc_) were measured on CT data, using either non-enhanced or, when available, contrast-enhanced CT scans. The readers were blinded to the clinical information of the patients.

Measurements of CT data were performed by the same two readers, Reader 1, >8 years, and Reader 2, >2 years of experience in CT imaging using dedicated software (Visage Client Software version 7.1.19, San Diego, CA, USA).

### Therapy

For prednisolone, the following standard scheme was used: 60 mg per day for six weeks, followed by a reduction of 10 mg per day per week until reaching 20 mg per day, then 15 mg per day for two weeks, 10 mg per day for four weeks, 7.5 mg per day for eight weeks, and a maintenance dose of 5 mg per day. In this scheme at later stages, deviations may have occurred due to side effects. For rituximab a standard dose of 750 mg was used.

### Disease progression

Metabolic progression between FU1 and FU2 was defined as an increase of at least 30% of the peak standardized uptake value corrected for lean body mass (SUL_peak_), adapted from PERCIST 1.0 criteria [23, 24], ensuring a standardized evaluation of PET-derived metabolic activity.

The standardized uptake value normalized to lean body mass (LBM) was calculated using the formula:

$$\:SUL=SUV\:x\:\frac{LBM}{weight}$$

LBM was estimated via the Janmahasatian formula [25], with body mass index (BMI) computed as weight (kg) divided by height (m^2^).

For male subjects, LBM was determined as:

$$\:LBM=\frac{9270\:\times\:\:weight}{6680\:+\:216\:\times\:\:BMI}$$

and for female subjects as:

$$\:LBM=\frac{9270\:\times\:\:weight}{8780+\:244\:\times\:\:BMI}$$

Patients who received rituximab between FU1 and FU2 were excluded from these analyses.

### Statistical analysis

Descriptive statistics of subjects were conducted. To evaluate the changes between examinations, relative changes of PET and CT metrics were computed as the difference in values of two exams divided by the value of the first exam and are expressed in percent of the BL value and reported as percentage. This reads for e.g. ∆SUV_max_:

$$\:\varDelta\:SUV=\frac{{SUVmax}_{follow-up}-\:{SUVmax}_{baseline}}{{SUVmax}_{baseline}}$$

To compare the differences in relative PET and CT parameter changes between the therapy groups, we initially performed a Kruskal-Wallis test. Following a significant result, pairwise comparisons between groups were conducted using the Mann-Whitney U test. Bonferroni correction was applied to adjust for multiple comparisons. To evaluate the therapeutic response of PET and CT parameters, the Wilcoxon signed-rank test for paired samples was used.

The Spearman rank correlation coefficient was used to examine the correlation between the PET and CT parameters.

For prediction of disease progression, multivariate logistic regression with a backward elimination approach was used. Pairwise comparisons between progression and non-progression groups were performed using the Mann-Whitney U test. P-values below 0.05 were considered statistically significant. Computations were primarily done using SPSS 29.0.0.0 (IBM Corporation, Armonk, NY, United States).

## Results

### Subjects

A total of 154 [^18^F]FDG PET/CT scans were evaluated. 50 patients (15 female) received at least two [^18^F]FDG PET/CT scans (BL and FU1), 36 patients received a FU2-, and 18 a FU3-PET/CT scan. The mean age of patients at the BL scan was 59 (range 41–78) years. Mean age of female patients at BL was 59.9 (range 46–78) years and of male patients was 59.3 (range 41–75) years. Mean weight was 82.5 (range 50.0–126) kg at BL. Mean time interval between BL and FU1 was 209.1 (range 104–477) days (median 202.5 days), and 234.9 (range 77–941) days between FU1 and FU2 (median 181.5 days), and 289.4 (range 137–978) days between FU2 and FU3 follow-up (median 203.5 days).

### Baseline characteristics

Before therapy in BL exams, the combination therapy group showed the highest means of SUV_max_ (10.5, range 7.5–13.8), SUV_mean_ (4.2, range 2.9–5.6) and SUV_peak_ (6.7, range 4.5–9.8), the prednisolone monotherapy group showed highest MAV (128.0, range 3.1–593.6) milliliters (ml). Detailed subgroup metrics at BL are shown in Table 1.

**Table 1:** Metrics of subgroups at baseline

	SUV_max_	SUV_mean_	SUV_peak_	MAV (ml)	CT_rim_ (mm)	CT_cc_ (mm)
Rituximab (*n* = 11)						
Mean	9.06	3.87	6.08	69.08	9.9	111.9
Standard Deviation	2.95	1.02	2.20	80.43	3.9	34.3
Minimum	4.4	2.6	3.0	7.9	4	55
Maximum	13.5	5.6	9.6	292.9	16	171
Prednisolone (*n* = 23)						
Mean	7.47	3.32	5.38	128.05	14.9	136.5
Standard Deviation	2.74	0.64	2.35	156.22	7.4	40.1
Minimum	2.9	2.4	2.4	3.1	5	63
Maximum	12.8	4.8	11.3	593.6	31	207
Prednisolone + Rituximab (*n* = 16)						
Mean	10.54	4.19	6.71	123.11	16.5	129.4
Standard Deviation	1.98	0.82	1.70	57.12	5.5	36.8
Minimum	7.5	2.9	4.5	73.5	10	69
Maximum	13.8	5.6	9.8	286.5	28	208

*SUV* Standard uptake value, *MAV* metabolic active volume, *CT* computed tomography, *ml* milliliters, *mm* millimeters, *cc* craniocaudal

Before the first [^18^F]FDG PET/CT scan, 14 patients have been treated with rituximab (*n* = 1), azathioprine (*n* = 1), prednisolone (*n* = 10), a combination of prednisolone + cyclophosphamide + rituximab + zanubrutinib (*n* = 1) and prednisolone + azathioprine (*n* = 1).

### Correlation between PET and CT parameters

From BL to FU3, all PET parameters significantly correlated with CT_rim_ (*p* ≤ 0.039). Regarding CT_cc_, significant correlations with all PET parameters were shown in all three follow-up examinations, while at BL only with MAV (*p* < 0.001). Details are shown in Table 2.

**Table 2:** Spearman correlation between PET and CT parameters

		SUV_max_	SUV_mean_	SUV_peak_	MAV
		Baseline			
Correlation Coefficient rho	CT_rim_	0.387	0.293	0.418	0.680
Significance p		0.005	0.039	0.003	< 0.001
rho	CT_cc_	0.074	−0.119	0.166	0.520
p		0.611	0.412	0.249	< 0.001
		First follow-up			
rho	CT_rim_	0.474	0.462	0.358	0.420
p		< 0.001	< 0.001	0.011	0.002
rho	CT_cc_	0.403	0.332	0.351	0.534
p		0.004	0.018	0.012	< 0.001
		Second follow-up			
rho	CT_rim_	0.443	0.560	0.492	0.368
p		0.007	< 0.001	0.002	0.027
rho	CT_cc_	0.473	0.534	0.486	0.438
p		0.004	< 0.001	0.003	0.007
		Third follow-up			
rho	CT_rim_	0.805	0.741	0.888	0.769
p		< 0.001	< 0.001	< 0.001	< 0.001
rho	CT_cc_	0.648	0.610	0.647	0.534
p		0.004	0.007	0.004	0.023

*SUV* Standard uptake value, *MAV* metabolic active volume, *CT* computed tomography, *mm* millimeters, *cc* craniocaudal

### Therapy between baseline and first follow-up

Between BL and FU1, 11 patients received rituximab monotherapy, 23 prednisolone monotherapy and 16 received combination therapy. In the rituximab group, three patients received one application, seven patients received two applications, and one patient received three applications. In the combination group, six patients received one application, and ten patients received two applications of rituximab.

Highest mean relative changes between BL and FU1 in PET and CT parameters have been seen in the combination therapy group (ΔSUV_max_ −63.4%, ΔSUV_mean_ −34.7%, ΔSUV_peak_ −57.6%, ΔMAV − 93.1%, ΔCT_rim_ −48.1%, ΔCT_cc_ −32.7%). Details are shown in Table 3.

**Table 3:** Relative changes of PET and CT parameters by therapy groups between baseline and first follow-up

	ΔSUV_max_	ΔSUV_mean_	ΔSUV_peak_	ΔMAV (ml)	ΔCT_rim_ (mm)	ΔCT_cc_ (mm)
Rituximab (*n* = 11)						
Mean	−45.572	−29.539	−36.984	−57.459	−34.468	−16.782
Std. Deviation	34.066	32.442	34.843	45.853	37.667	38.243
Minimum	−100.00	−100.00	−100.00	−100.00	−100.00	−100.00
Maximum	15.91	11.11	9.52	34.55	40.00	48.35
Prednisolone (*n* = 23)						
Mean	−38.865	−21.820	−37.569	−47.330	−37.349	−12.600
Std. Deviation	29.998	21.862	35.404	118.639	22.934	25.405
Minimum	−100.00	−100.00	−100.00	−100.00	−100.00	−100.00
Maximum	41.38	12.00	75.86	465.85	−5.26	31.75
Rituximab + Prednisolone (*n* = 16)						
Mean	−63.417	−34.653	−57.551	−93.108	−48.060	−32.676
Std. Deviation	16.692	25.811	20.780	13.717	36.694	31.726
Minimum	−100.00	−100.00	−100.00	−100.00	−100.00	−100.00
Maximum	−34.67	20.00	−19.61	−55.37	18.18	5.83

*SUV* Standard uptake value, *MAV* metabolic active volume, *CT* computed tomography, *ml* milliliters, *mm* millimeters, *cc* craniocaudal

**Fig. 1:**
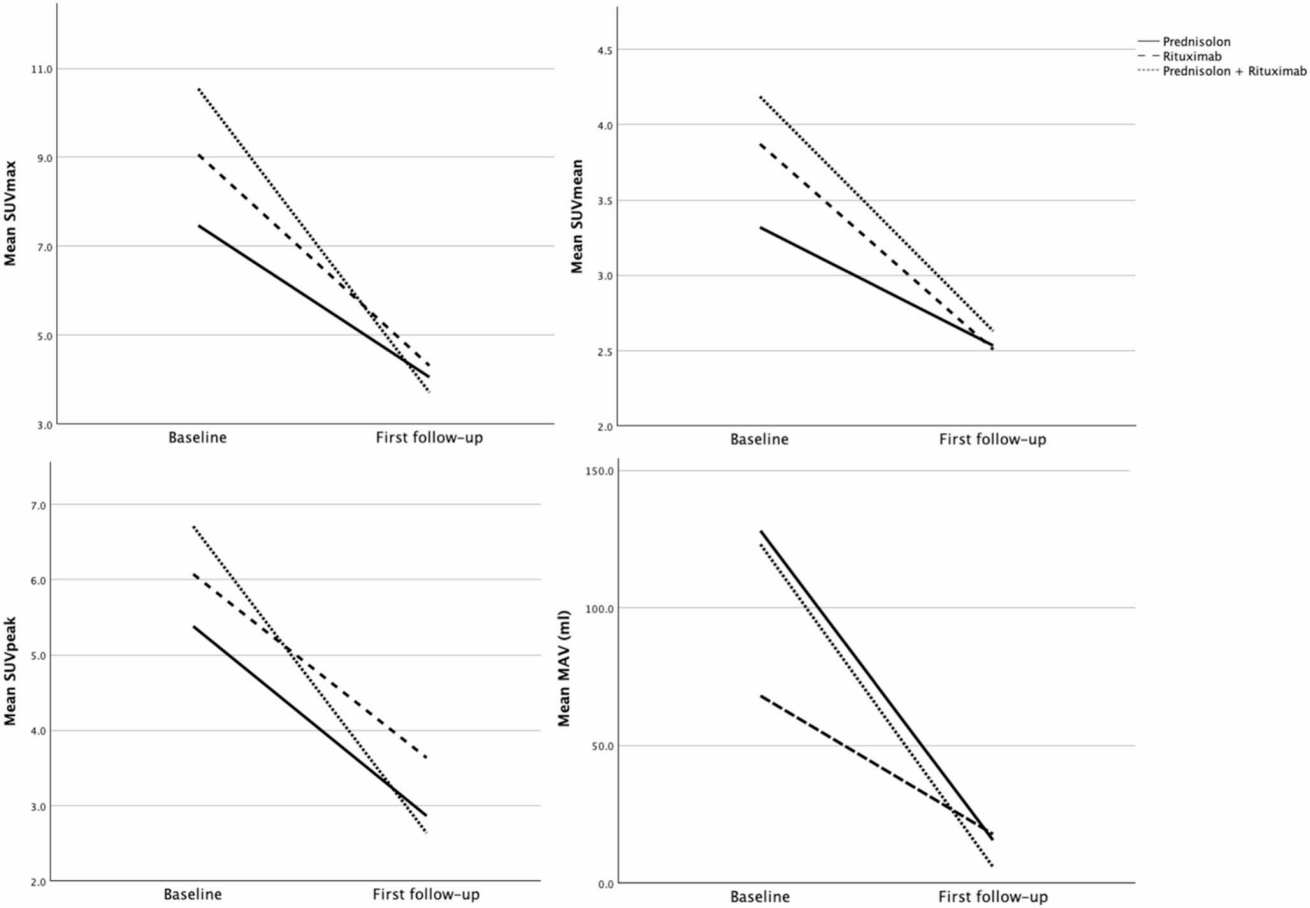
Therapy response of PET parameters between baseline and first follow-up (*n* = 50; mean interval 209.1 (range, 104–477) days). SUV *Standard uptake value*, MAV *metabolic active volume*, ml *milliliters*

All PET and CT parameters decreased significantly (*p* ≤ 0.021) except for CT_cc_ in the subgroup treated with rituximab (*p* = 0.113). Results for each therapy group are summarized in Table 4 and visualized in Fig. 1.

**Table 4:** Therapy response of PET and CT parameters (Wilcoxon signed-rank test) by therapies between baseline and first follow-up

	SUV_max_	SUV_mean_	SUV_peak_	MAV (ml)	CT_rim_ (mm)	CT_cc_ (mm)
Rituximab (*n* = 11)						
Z	−2.669	−2.401	−2.312	−2.667	−2.540	−1.584
Significance p	0.008	0.016	0.021	0.008	0.011	0.113
Prednisolone (*n* = 23)						
Z	−3.954	−3.860	−3.620	−3.741	−4.205	−2.938
p	< 0.001	< 0.001	< 0.001	< 0.001	< 0.001	0.003
Rituximab + Prednisolone (*n* = 16)						
Z	−3.517	−3.362	−3.517	−3.516	−3.239	−3.361
p	< 0.001	< 0.001	< 0.001	< 0.001	0.001	< 0.001

*SUV* Standard uptake value, *MAV* metabolic active volume, *CT* computed tomography, *ml* milliliters, *mm* millimeters, *cc* craniocaudal

Relative changes in PET and CT parameters differed significantly between therapy groups in SUV_max_ and MAV (H = 9.758, *p* = 0.008; H = 14.478, *p* < 0.001), and CT_cc_ (H = 6.257, *p* = 0.044). Post-hoc analysis showed that the prednisolone and combination therapy groups differed in all PET parameters and CT_cc_ (*p* ≤ 0.043), while the rituximab and combination therapy group only differed in MAV (*p* = 0.002). No significant differences were found between the monotherapy groups. Results by each therapy group are shown in Table 5.

**Table 5:** Comparison of relative changes of PET and CT parameter between therapy groups

	ΔSUV_max_	ΔSUV_mean_	ΔSUV_peak_	ΔMAV	ΔCT_rim_	ΔCT_cc_
Prednisolone vs. Rituximab						
Mann-Whitney U	97.500	107.000	123.500	113.500	123.500	119.500
Z	−1.068	−0.718	−0.110	−0.479	−0.111	−0.258
Significance p	0.291	0.490	0.913	0.637	0.913	0.800
Rituximab vs. Rituximab + Prednisolone						
Mann-Whitney U	53.500	77.500	53.500	27.000	70.500	59.500
Z	−1.703	−0.519	−1.703	−3.012	−0.864	−1.407
p	0.089	0.610	0.089	0.002	0.394	0.162
Prednisolone vs. Rituximab + Prednisolone						
Mann-Whitney U	76.500	106.000	113.500	63.000	148.000	94.000
Z	−3.069	−2.228	−2.013	−3.455	−1.029	−2.570
p	0.002	0.026	0.043	< 0.001	0.315	0.009

*SUV* Standard uptake value, *MAV* metabolic active volume, *CT* computed tomography, *mm* millimeters, *cc* craniocaudal

In the rituximab monotherapy group, significant decreases were observed only after two doses of SUV_max_, SUV_mean_, MAV, CT_rim_ and CT_cc_ (*p* = 0.028–0.046) with a strong trend towards improvement also in SUV_peak_ (*p* = 0.063), while after application of only one dose there was no significant response (*p* = 0.109-1.000). The combination therapy group showed the strongest response after two doses across all parameters (*p* ≤ 0.007). Also, significant response after application of one dose was seen in SUV_max_ (*p* = 0.028), SUV_peak_ (*p* = 0.028), MAV (*p* = 0.028) and CT_cc_ (*p* = 0.046) with numeric improvement in SUV_mean_ (*p* = 0.075) and CT_rim_ (*p* = 0.066). Mean relative reduction of SUV_max_ was 55.2% after one application and 76.5% after two applications of rituximab.

### Therapy between first and second follow-up

Between first and second follow-up, six patients received further medication: Four patients received one and two patients received two applications of rituximab with a significant decrease only of SUV_max_ (Z=−2.201, *p* = 0.028) while the other parameters showed moderate response: SUVmean (Z=−1.753, *p* = 0.080), SUV_peak_ (Z=−1.782, *p* = 0.075), MAV (Z=−0.134, *p* = 0.893) and CT_rim_ (Z=−1.753, *p* = 0.080), CT_cc_ (Z=−1.483, *p* = 0.138).

### Progression

At FU2 eight patients showed disease progression with 22 patient showing remission or stable disease. In the progression group, the mean time between BL and FU1 was 213.4 (median 195.0) days, and between FU1 and FU2 226.0 (median 201.5) days. In the non-progression group, the mean time interval from BL to FU1 was 190.9 (median 187.0) days, and from FU1 to FU2 207.7 (median 178.0) days. These follow-up durations did not differ significantly between the two groups (BL–FU1: *p* = 0.291; FU1–FU2: *p* = 0.241). For the prediction of disease progression, multivariate logistic regression revealed highest significance for MAV and CT_rim_ before (*p* = 0.080 and *p* = 0.064) and after backward elimination (*p* = 0.041 and 0.046). Patients with disease progression showed higher MAV at BL (Z=−1.172, *p* = 0.241) compared to patients without progression (217.33 ml versus 100.86 ml), while differences in CT_rim_ were weak (Z=−0.305; *p* = 0.760; 13.8 mm versus 15.1 mm). All other PET and CT parameters at BL did not differ significantly between patients with and without progression. Details are shown in Table 6.

**Table 6:** Metrics of baseline PET parameters by disease progression between first and second follow-up

Baseline PET parameters	Disease progression	N	Mean	Standard deviation	Logistic regression	Mann-Whitney U test	
					Significance *p*	Z	Significance *p*
SUV_max_	no	22	9.08	2.98	0.206	−0.305	0.760
	yes	8	8.76	2.55			
SUV_mean_	no	22	3.79	0.90	0.706	−0.023	0.981
	yes	8	3.65	0.67			
SUV_peak_	no	22	6.04	2.21	0.201	−0.540	0.589
	yes	8	6.45	1.88			
MAV (ml)	no	22	100.86	78.42	0.080	−1.172	0.241
	yes	8	217.33	211.52			
CT_rim_ (mm)	no	22	15.1	7.1	0.064	−0.305	0.760
	yes	8	13.8	5.6			
CT_cc_ (mm)	no	22	123.6	41.0	0.459	−1.196	0.232
	yes	8	140.5	38.2			

*SUV* Standard uptake value, *MAV* metabolic active volume, *CT* computed tomography, *ml* milliliters, *mm* millimeters, *cc* craniocaudal

**Fig. 2:**
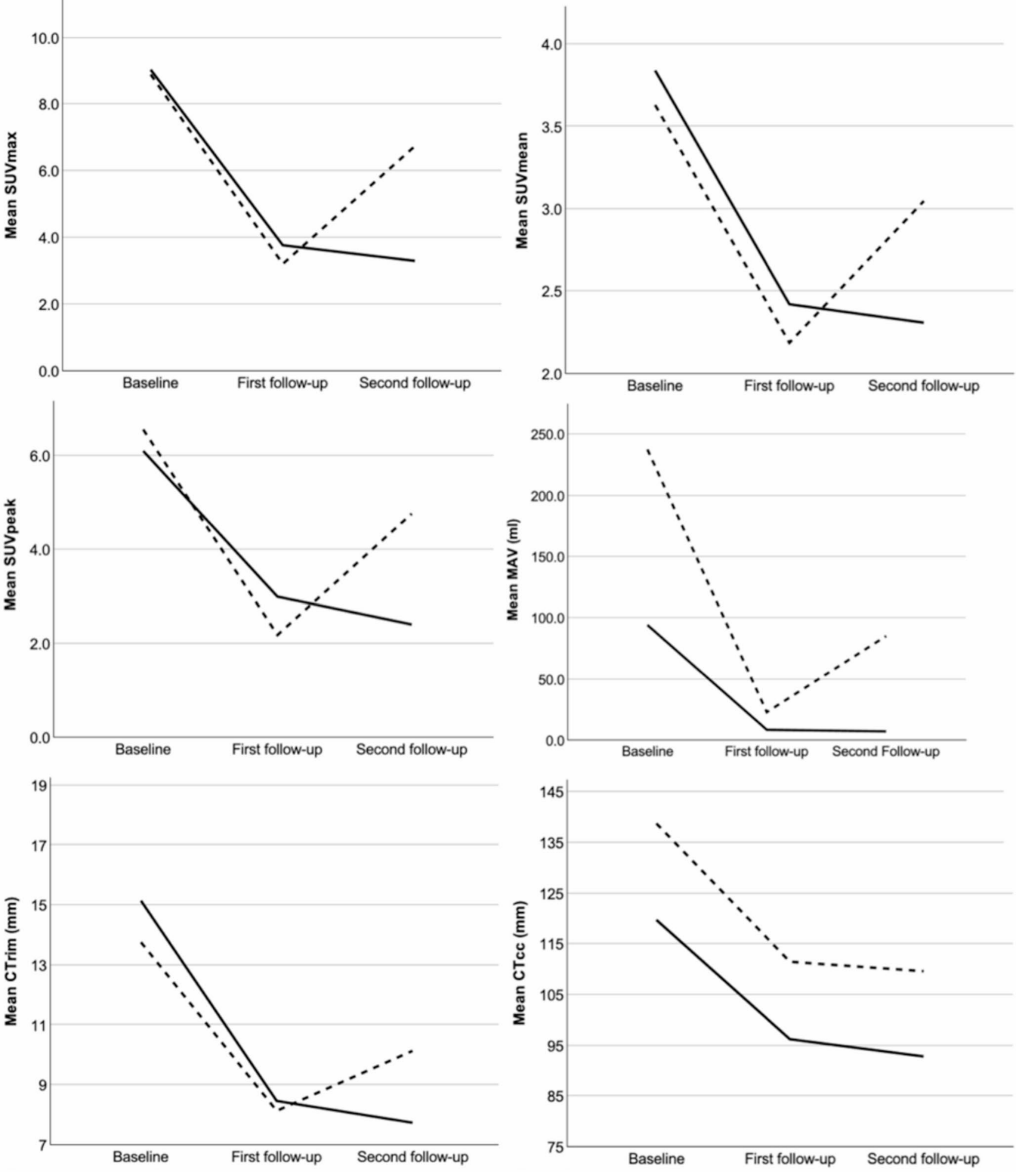
Longitudinal progression of PET and CT by relapse (dashed line) and no relapse (solid line) between baseline, first (*n* = 50) and second follow-up (*n* = 34). SUV *Standard uptake value*, MAV *metabolic active volume*, CT *computed tomography*, ml *milliliters*, mm *millimeters*, cc *craniocaudal*

CT_rim_ and CT_cc_ at FU2 did not differ significantly between progression and non-progression group (*p* = 0.831 and *p* = 0.453, respectively), while SUV_max_ (*p* = 0.004), SUV_peak_ (*p* < 0.001) and MAV (*p* = 0.009) showed strong differences. Relative progression between FU1 and FU2 differed strongly in ΔSUV_max_ (Z=−2.268, *p* = 0.023), ΔSUV_mean_ (Z=−2.685, *p* = 0.007), ΔSUV_peak_ (−3.874, *p* < 0.001) and weak in ΔMAV (Z=−1.012, *p* = 0.312), ΔCT_rim_ (Z=−0.640, *p* = 0.522) and ΔCT_cc_ (Z=−0.830, *p* = 0.407). Longitudinal progression of PET and CT parameters from BL to FU2 is shown in Fig. 2.

Within the progression group, two patients received rituximab (both treated with prednisolone before BL [^18^F]FDG PET/CT), four patients received prednisolone, and two patients received the combination therapy between BL and FU1. In the prednisolone group statistical difference between patients with progression (*n* = 4) and non-progression (*n* = 12) was highest of all parameters (*p* = 0.182) in MAV, but only moderate in logistic regression (*p* = 0.174).

**Fig. 3:**
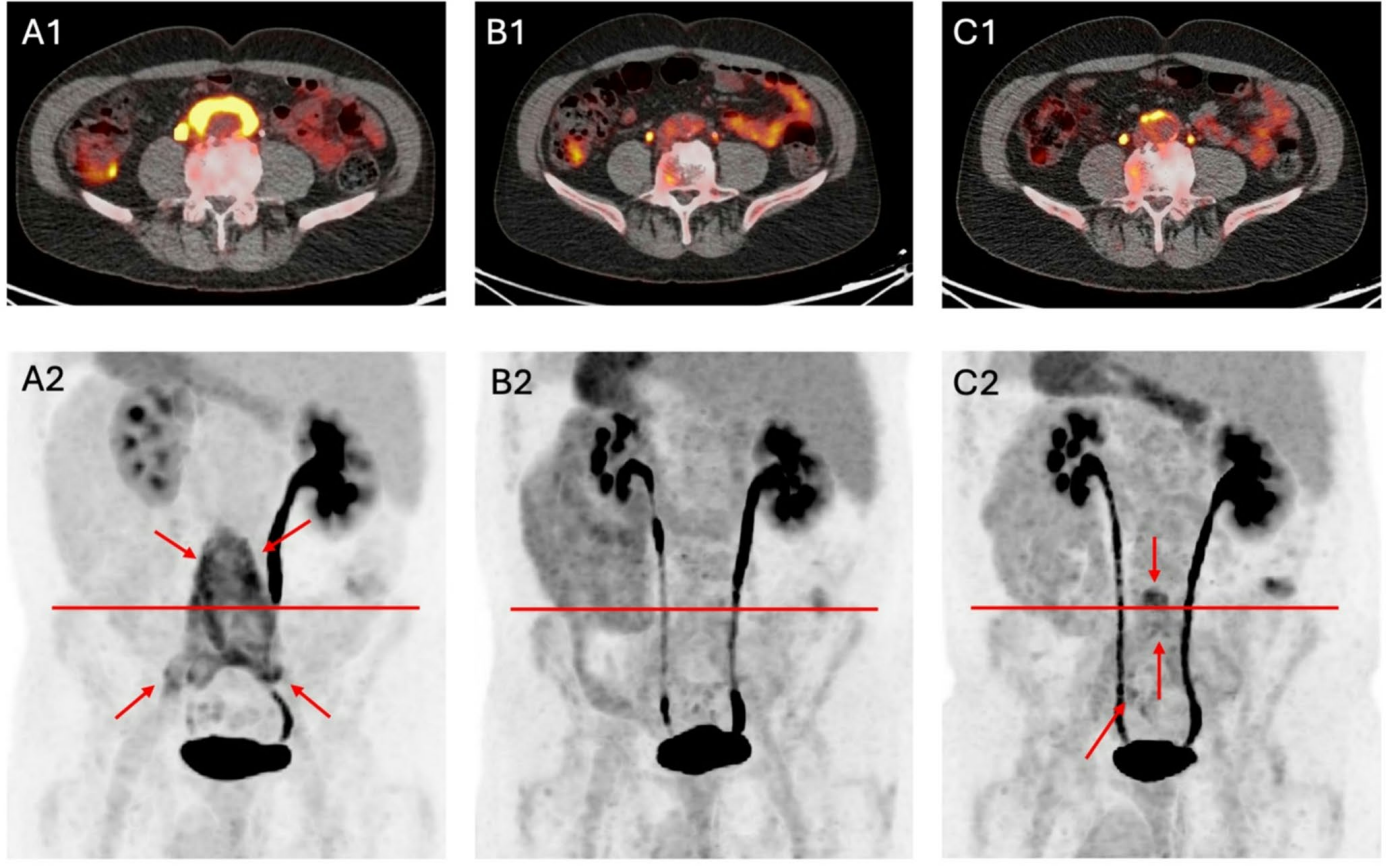
Course of retroperitoneal fibrosis in a patient showing progression between first and second follow-up. A1, B1, and C1 display axial 18 F-FDG PET/CT fusion images, with corresponding red lines in the maximum intensity projection (MIP) images (A2, B2, and C2). Red arrows delineate findings in A2 and C2. The corresponding PET values at baseline, first and second follow-up are as follows: SUV_max_: 11.9 vs. 3.5 vs. 7.5, SUV_mean_: 4.4 vs. 2.8 vs. 2.9, SUV_peak_: 9.1 vs. 2.6 vs. 4.5, and metabolic active volume (MAV): 180.5 vs. 4.6 vs. 67.7 milliliters

An example of the course of disease between BL and 2FU is shown in Fig. 3. In this case, between BL and FU1 all PET and CT parameters decreased, and between FU1 and FU2 all PET parameters increased, while CT_rim_ and CT_cc_ decreased (−28.6% and − 4,8%).

## Discussion

In Retroperitoneal Fibrosis imaging plays a crucial role as a rare and often silent inflammatory disease that usually becomes apparent only through complications, making the detection of disease activity and treatment monitoring particularly challenging.

### Treatment response

In our study, all PET parameters showed significant correlations with CT_rim_ across all timepoints (BL, FU1, FU2), indicating a consistent link between metabolic and morphologic disease activity throughout treatment, with SUV_max_, SUV_peak_, and MAV showing the strongest associations with morphologic parameters.

Therapy response analysis revealed significant reductions in all PET parameters and CT_rim_ from BL to FU1, most pronounced in the combination therapy group, emphasizing its superior efficacy by targeting multiple inflammatory pathways simultaneously. Both monotherapies also achieved significant metabolic and morphologic improvements, except for CT_cc_ in the rituximab group, confirming their individual effectiveness. Patients treated with rituximab monotherapy also exhibited significant reductions in PET parameters, suggesting that this therapy contributes to metabolic improvements. However, this should be interpreted with caution, as all but one patient in the rituximab group had received some form of therapy before BL. Still, our findings support former studies with prednisolone as concurrent, dose-reduced therapy or as pre-medication [16–18, 20].

Notably, significant responses were seen after two applications of rituximab and even after one application in combination therapy. However, comparability to previous studies is limited due to differences in dosing (1000 mg vs. 750 mg) and administration intervals [16, 18–20].

In our study, treatment allocation was not randomized but based on clinical presentation and [^18^F]FDG PET/CT findings at BL, with the decision made by the treating nephrologist. This individualized approach reflects routine clinical practice in RPF, especially given the frequent need for rapid treatment initiation in high-risk patients. However, it may have contributed to baseline differences in disease burden between therapy groups and limits the comparability of outcomes. In addition, the limited sample size further constrains the generalizability of our findings. Prospective, standardized treatment studies will be essential to confirm these observations and reduce selection-related bias.

### Disease progression

Following successful initial therapy, systematic follow-up is essential to enable early detection of disease progression or recurrence. Accordingly, the identification of a reliable prognostic baseline parameter to guide early therapeutic decision-making would be of great relevance.

In our study, between FU1 and FU2 eight patients showed metabolic progression. Notably, BL MAV was higher in patients who later showed disease progression, while other PET and CT values did not differ significantly between these two groups.

Since MAV is derived from the lesion volume, its interpretation must account for morphological parameters. Although our study did not explicitly assess lesion volume, logistic regression analysis indicated that BL CT_rim_ had moderate predictive value for progression. However, the Mann-Whitney U test did not show strong differences between patients with progression and without. Future studies should correlate MAV with the exact lesion volume to further evaluate whether this represents a purely volumetric phenomenon or reflects the actual metabolically active tumor burden.

In our study, progression was defined solely as metabolic progression, and clinical parameters were not included, as even subtle new or increasing metabolic activity may justify early intervention to prevent complications. Nevertheless, our findings - although derived from an inflammatory disease context - are in line with oncological studies demonstrating that pre-therapeutic metabolic tumor volume is an independent prognostic parameter [26, 27]. This suggests that MAV might serve as a prognostic parameter for predicting disease progression in follow-up examinations also in RPF, highlighting the potential benefit of routinely including MAV in BL reports instead of relying solely on SUV_max_ and SUV_peak_.

In this context, it is worth mentioning that four of eight patients with disease progression received prednisolone monotherapy as therapy between BL and FU1, while two patients each were treated with rituximab monotherapy or in combination. Although no conclusive statement can be made due to small sample size, this observation raises the possibility that prednisolone as monotherapy may be associated with higher risk of progression, whereas rituximab and the combination therapy might help prevent progression.

In the assessment of progression at FU2, it is noteworthy that, despite significant correlations between PET parameters and CT_rim_ at all time points, most PET parameters demonstrated significant differences between the progression and non-progression groups, whereas CT_rim_ and CT_cc_ did not exhibit any statistically significant variation. This suggests that PET imaging provides superior discriminatory power for detecting disease progression. Nevertheless, due to the marked therapeutic response observed on CT between baseline and FU1, CT can be considered a suitable modality for initial therapy monitoring. However, in the course of follow-up - particularly for the detection of progression - it should be complemented by PET imaging.

### Limitations

When interpreting our measurements, it is crucial to consider potential sources of bias and limitations that may impact accuracy: The measurement of the cranio-caudal extension CT_cc_ of lesions appeared to be challenging, especially of small residuals in non-contrast-enhanced scans. This limitation may have influenced the accuracy of CT_cc_ assessments and, consequently, the evaluation of therapy effects on structural disease changes. In addition, morphologic measurements were based on both non-enhanced and, when available, contrast-enhanced CT scans, as the use of intravenous contrast agents was often contraindicated due to the high prevalence of renal impairment in patients with RPF. Nevertheless, these retroperitoneal masses typically appear well demarcated against the surrounding retroperitoneal fat, allowing for reliable visual identification even in non-enhanced scans. When delineating lesions in PET scans, it is important to account for the fact that the aortic wall commonly shows FDG uptake [28] or surrounding organs such as the ureters, which can lead to an overestimation of MAV. To mitigate this and ensure that only the relevant active areas with exclusion of the blood pool are detected, we used SUV_mean_liver_ as the lower absolute threshold in our measurements, and excluded surrounding organs, as best as possible. This approach was also chosen to minimize scanner-related variability. Notably, both scans on the non-digital system were conducted during follow-up, so all BL scans used for prognostic modeling were acquired on the same PET/CT scanner ensuring consistency in quantitative assessment at BL.

We considered the BL [^18^F]FDG PET/CT scan as the first available scan before therapy. However, a limitation is that 14 of 50 patients had received treatments before their BL [^18^F]FDG PET/CT scan, most of which were administered in external centers and were often incompletely documented. As far as documented, these treatments were administered several months before the PET imaging, minimizing potential confounding effects. During or after initial therapy, most patients received low-dose prednisolone with irregular adjustments of dosages - often by external physicians - resulting in incomplete documentation.

## Conclusion

The results of this study underscore the superiority of [^18^F]FDG PET/CT against CT in therapy monitoring, especially in detection and prediction of disease progression at follow-ups. BL MAV might serve as a parameter for the prediction of progression, which highlights the importance of considering a broader range of PET parameters beyond the commonly used SUV_max_ or SUV_peak_ at BL and follow-ups to guide therapy decision and adjust follow-up intervals.

Still, especially when PET is not readily available, CT can be considered a valuable tool for initial therapy monitoring.

## Data Availability

The datasets generated during and/or analyzed during the current study are available from the corresponding author on reasonable request.
